# MRI-Based Radiomics for Differentiating Orbital Cavernous Hemangioma and Orbital Schwannoma

**DOI:** 10.3389/fmed.2021.795038

**Published:** 2021-12-16

**Authors:** Liang Chen, Ya Shen, Xiao Huang, Hua Li, Jian Li, Ruili Wei, Weihua Yang

**Affiliations:** ^1^Department of Ophthalmology, Shanghai Changzheng Hospital, Shanghai, China; ^2^Department of Imaging, Shanghai Changzheng Hospital, Shanghai, China; ^3^Affiliated Eye Hospital, Nanjing Medical University, Nanjing, China

**Keywords:** orbit, radiomics, cavernous hemangioma, schwannoma, machine learning

## Abstract

**Aim:** The purpose of this work was to develop and evaluate magnetic resonance imaging (MRI)-based radiomics for differentiation of orbital cavernous hemangioma (OCH) and orbital schwannoma (OSC).

**Methods:** Fifty-eight patients (40 OCH and 18 OSC, confirmed pathohistologically) screened out from 216 consecutive patients who presented between 2015 and 2020 were divided into a training group (28 OCH and 12 OSC) and a validation group (12 OCH and 6 OSC). Radiomics features were extracted from T1-weighted imaging (T1WI) and T2-weighted imaging (T2WI). *T*-tests, the least absolute shrinkage and selection operator (LASSO), and principal components analysis (PCA) were used to select features for use in the classification models. A logistic regression (LR) model, support vector machine (SVM) model, decision tree (DT) model, and random forest (RF) model were constructed to differentiate OCH from OSC. The models were evaluated according to their accuracy and the area under the receiver operator characteristic (ROC) curve (AUC).

**Results:** Six features from T1WI, five features from T2WI, and eight features from combined T1WI and T2WI were finally selected for building the classification models. The models using T2WI features showed superior performance on the validation data than those using T1WI features, especially the LR model and SVM model, which showed accuracy of 93% (85–100%) and 92%, respectively, The SVM model showed high accuracy of 93% (91–96%) on the combined feature group with an AUC of 98% (97–99%). The DT and RF models did not perform as well as the SVM model.

**Conclusion:** Radiomics analysis using an SVM model achieved an accuracy of 93% for distinguishing OCH and OSC, which may be helpful for clinical diagnosis.

## Introduction

Orbital cavernous hemangioma (OCH) is a common primary tumor representing ~8% of all orbital lesions (1). Patients with OCH typically show slow-moving progression and painless proptosis, although some suffer from disturbance in vision and visual fields (2). Though having a similar clinical manifestation to OCH (3), orbital schwannoma (OSC) accounts for <1% of orbital lesions. However, the prognosis and therapeutic strategies for the two tumors are always different. Observation is a possible choice for those patients newly diagnosed with OCH, but surgical intervention is often needed for OSC patients, as OSC typically shows progressive growth (4, 5). Therefore, it is necessary to identify the two tumors (3, 6, 7).

Because of their similar clinical features, the identification of OCH and OSC can be clinically challenging. Several imaging studies attempted to clarify the differences between them (8–10), indicating that OCH has a more regular shape than OSC, and that markedly homogeneous hyperintense signal on T2-weighted magnetic resonance imaging (MRI) favors OCH rather than OSC. Furthermore, the contrast enhancement on dynamic contrast-enhanced MRI may also be helpful for distinguishing the two tumors; OCH shows “progressive” enhancement starting from a small point or portion, with the contrast media later filling up the tumor, whereas OSC shows enhancement starting from a wide area, with heterogeneous or homogeneous enhancement occurring later (8). However, these findings may not always work well in the clinic, with some images being indistinguishable and dividing opinion with an absence of objective evidence. Therefore, an objective identification method would be preferred by the clinician.

Radiomics, a promising and rapidly growing discipline, can be defined as the quantification of the phenotypic features of a lesion from medical images. It involves the extraction of a large number of quantitative features from medical images and their subsequent analysis to support clinical decision-making (11–13). It can overcome some of the limitations of subjective analysis with the human eye, squeezing out more information from each image (14). Radiomics approaches are currently becoming more and more popular in clinical auxiliary diagnosis and prognosis (15–17).

However, to the best of our knowledge, there is no published study using radiomics to distinguish OCH from OSC. In this study, we applied radiomics analysis to the differentiation of the two tumors and evaluated the results.

## Subjects and Methods

### Patients

Two hundred and sixteen consecutive patients diagnosed with OCH or OSC between 2015 and 2020 were identified in the electronic medical record system (EMR) of Shanghai Changzheng Hospital. All patients were retrospectively recruited and signed informed consent before this study.

**Figure 1:**
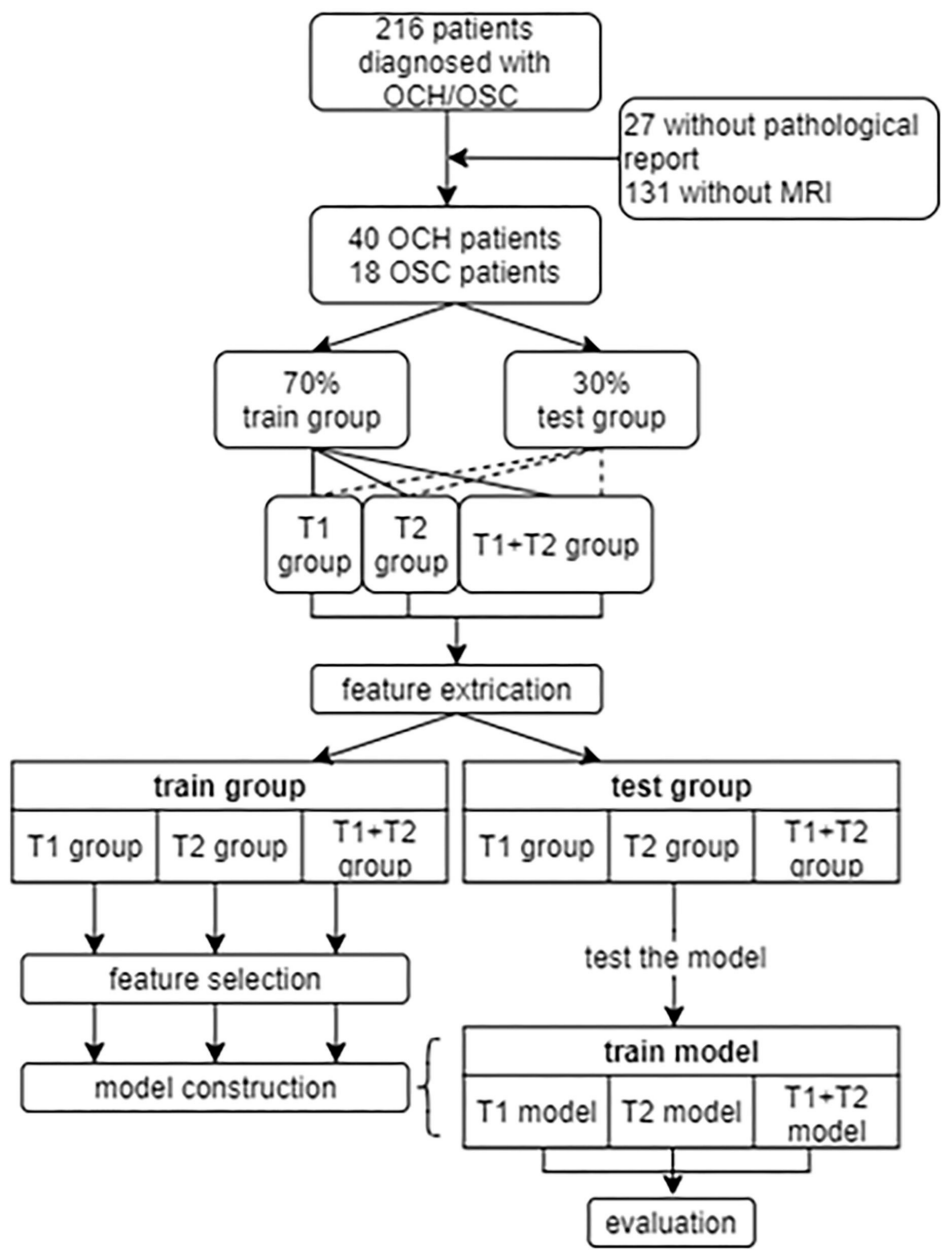
The recruitment of patients and design of this study. OCH, orbital cavernous hemangioma; OSC, orbital schwannoma.

The inclusion criteria were: (1) patients newly diagnosed with OCH or OSC with biopsy confirmation; (2) tumor involving the eye of only one side; (3) complete and clear MRI data collected on scanners of the same model; (4) no surgical or other therapy prior to MRI scanning. Patients with incomplete MRI or with imaging of insufficient quality were excluded. Figure 1 summarizes the patient recruitment process.

### MRI Pre-processing

The DICOM format MRI of all included patients were acquired from the picture archiving and communication system of Shanghai Changzheng Hospital. The MRI examinations were performed on a 3.0-T scanner [Achieva 3.0T (TX) DS MR system, Philips Healthcare] with heart of FreeWave, an advanced modular 32-channel digital data acquisition system, and high-resolution head coil. Pre-contrast turbo spin-echo (TSE) T1-weighted imaging (T1WI) and T2-weighted imaging (T2WI) with fat suppression were acquired. The parameters were set as follows, for T1WI, the repetition time (TR) was 470 ms, echo time (TE) 12 ms, 16 slicers with thickness of 3 mm and a gap of 0.3 mm, 200–400 mm field of view (FOA), the flip angle (FA) was 90° and the number of signal acquisition (NSA) was 1; for T2WI, the driven equilibrium technology was used and the TR 3,000 ms, TE 80 ms, 16 slicers with thickness of 4 mm and a gap of 0.4 mm, 200–400 mm FOA, FA 90° and NSA was 2. To compare the efficiency of the different sequences, radiomics analyses were performed separately on the T1WI, T2WI, and combined (T1WI + T2WI) sequences. For each sequence, ~70% (28 OCH and 12 OSC) of the acquisitions were selected as the training data and the remaining 30% formed the validation data.

First, MRI bias was corrected using the N4ITK MRI bias correction (18), then all images were horizontally mirrored. Regions of interest (ROIs) were then outlined on each slice by an ophthalmologist and a radiologist using the free open-source software package 3D Slicer version 4.11(https://download.slicer.org/). The ROIs for each patient were outlined separately in the original images and mirror images, to reduce bias. The intraclass correlation coefficients (ICCs) between the two researchers were calculated for all extracted features to determine the reliability of the ROIs.

### Feature Extraction and Selection

The feature extraction was performed for all selected MRIs and their corresponding ROI masks using Python 3.7 (https://www.python.org/downloads/release/python-3711/) with Official default parameters (http://www.radiomics.io/pyradiomicsnotebook.html). The features included first order features, shape features, gray level co-occurrence matrix (GLCM), gray level dependence matrix (GLDM), gray level run length matrix (GLRLM), gray level size zone matrix (GLSZM), and neighboring gray tone difference matrix (NGTDM). These features are defined in the Results section. Zero-mean normalization was applied to these quantitative features following deletion of null values and features stored as string type.

In this analysis, to avoid overfitting and balance the limited samples and redundant features, we adopted four methods to select features. After simple *t*-tests, the LASSO linear regression model was applied, a model that can avoid overfitting and is suitable for analyzing small samples with high-dimensional features (19). However, many features still remained after the application of LASSO, so we then applied recursive feature elimination-cross validation (RFE-CV), a feature selection method that iteratively removes the least important features until the optimal number is reached. Despite these measures, the results were still not significant. Considering the potential for collinearity among the features, we created a correlation heat map, which confirmed out suspicions. Therefore, the dimensionality reduction method of PCA was adopted, which works by recombining a new set of composite variables unrelated to each other from the original features, and a few of these composite variables were extracted to faithfully reflect the original features as much as possible. Finally, fewer features were selected to construct the models (six features on T1WI, five features on T2WI, eight features on T1 + T2). Statistical analysis and plotting were performed with R (R vision 4.0.3, https://cran.r-project.org/).

### Model Construction and Evaluation

The selected features were used to build the classification models. In this analysis, a logistic regression (LR) model, support vector machine (SVM) model (linear kernel), random forest (RF) model, and decision tree (DT) model were constructed. All four models were evaluated on the validation data according to their accuracy score and the area under the receiver operating characteristics (ROC) curve (AUC). A nomogram was also built to visualize a multiparametric MRI prognostication model using radiomics features. Statistical analyses were performed using R statistical software (R version 4.0.3). A *P*-value <0.05 was considered statistically significant.

## Results

### Patients and MRI

**Figure 2:**
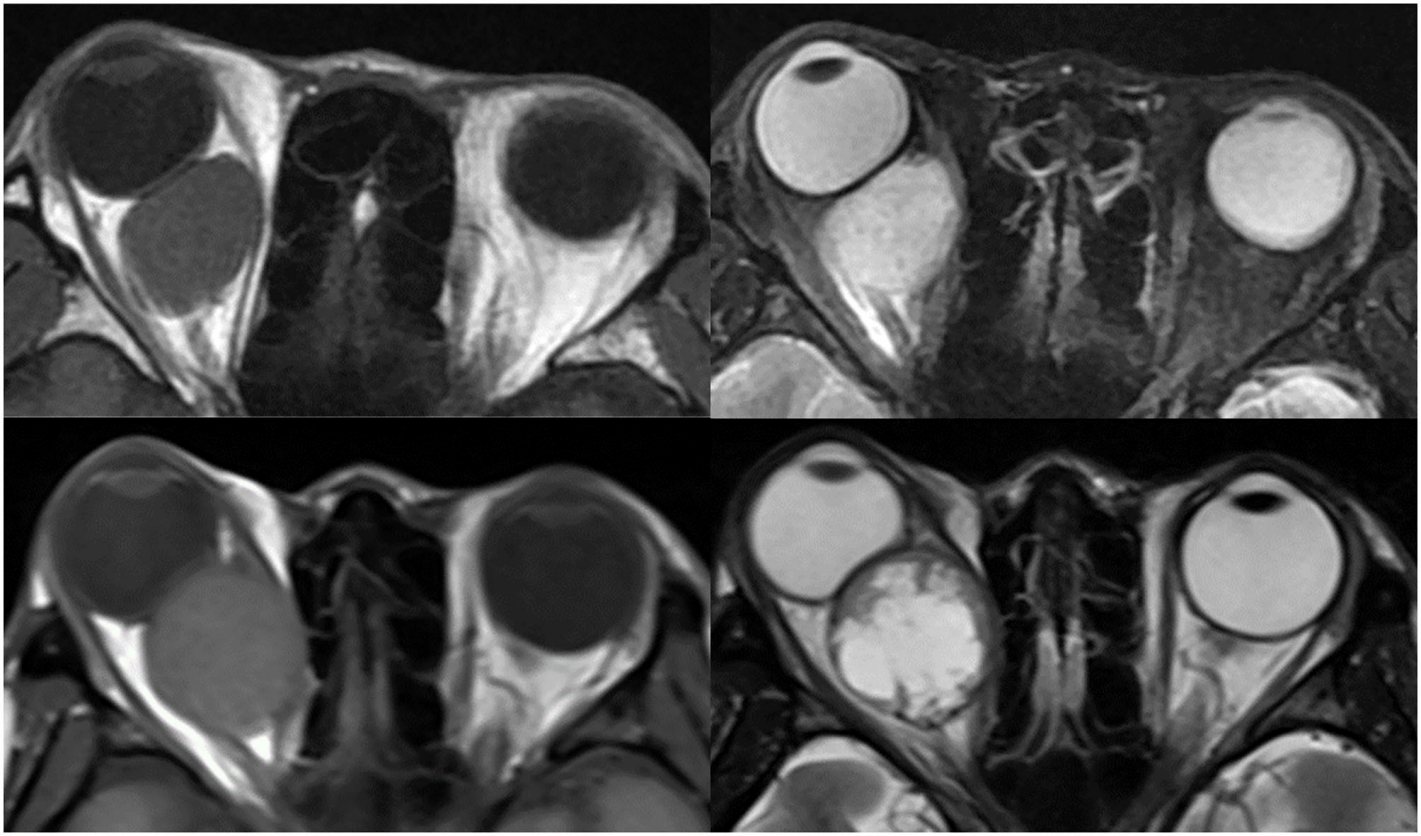
Example images of OCH and OSC. Top left is a T1-weighted image of a patient with OCH, top right is a T2-weighted image of the same patient. Bottom left is a T1-weighted image of a patient with OSC, bottom right is a T2-weighted image of the same patient.

As shown in Figure 1, 58 patients were eventually included in our study. From these patients, 1640 T1 or T2-weighted MR images in DICOM format were finally selected for further analysis. The lesions showed hypointensity on T1WI and hyperintensity on T2WI. Figure 2 shows example MR images. The demographic information of the included patients is shown in Table 1.

**Table 1:** Patient demographic information.

	**OCH** **(***n*** = 40)**	**OSC** **(***n*** = 18)**	***p*** **-value**
Gender			
Female	24 (60%)	8 (44%)	
Male	16 (40%)	10 (56%)	0.41
Age	49 (40–57)	47 (41–50)	0.62
Involvement			
Left	20 (50%)	8 (44%)	
Right	20 (50%)	10 (56%)	0.91
Intraconal	39	18	
Extraconal	1	0	
Tumor			
Size(mm^3^)	4776.07	7602.30	0.03
Sphericity	0.70 (0.69–0.74)	0.66 (0.64–0.69)	0.11
T1WI MEAN SIGNAL INTENSITY	573.43 (358.29–765.61)	711.15 (400.96–917.29)	0.30
T2WI mean signal intensity	662.62 (574.92–769.09)	792.05 (608.97–971.64)	0.15

*Tumor size was calculated based on the ROI. Tumor sphericity took values between 0 and 1, the higher the value (closer to 1), the closer to sphere the tumor. All values of tumor size, sphericity and signal intensity were from the features extracted*.

### Feature Extraction and Selection

A total of 107 features were selected from either T1 or T2 sequences for further analysis. These 107 features included 14 shape features, 18 first order features, 24 GLCMs, 14 GLDMs, 16 GLRLMs, 16 GLSZMs, and 5 NGTDMs. Of the above features, the shape features mainly describe the size and shape of the ROI and are only calculated for the non-derived image and mask, the first order features describe the distribution of voxel intensities within the image region defined by the mask and are computed using common basic metrics, and the remaining features describe texture and gray level intensity distributions with different algorithms and complexity. The ICCs of all extracted features were over 75%.

**Figure 3:**
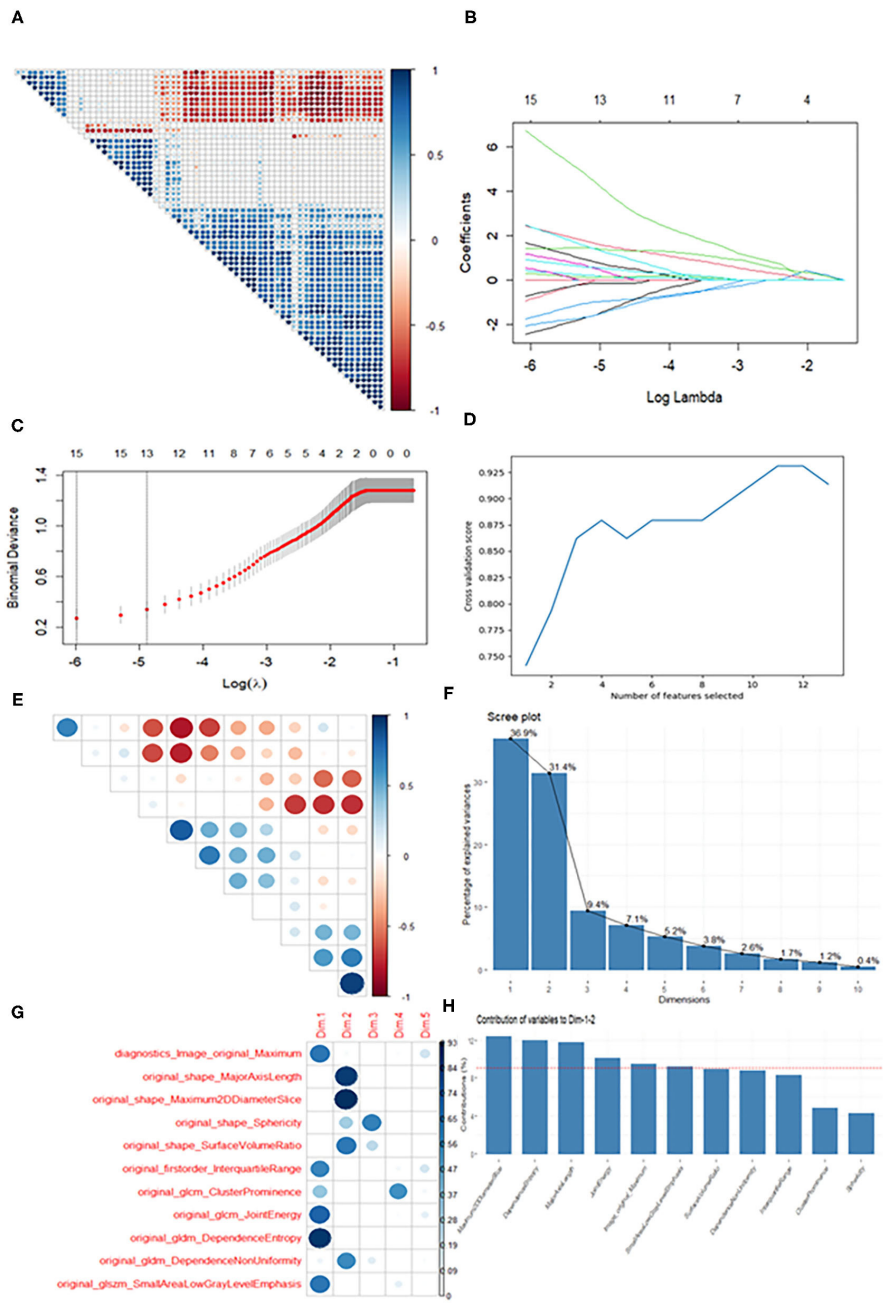
Diagram of the feature selection process. Images **(A,E)** are correlation heat maps demonstrating correlations between features. The deeper the color, the stronger the correlation. Red indicates negative correlation and blue positive correlation. Image **(B)** shows the results of the LASSO regression. With increasing penalty parameter the coefficients of the features approach zero and finally converge on an optimal solution. Image **(C)** shows the results of LASSO-CV. The right dotted line indicates the standard error of the minimum mean square error (the left dotted line) and corresponds to the feature number on the top coordinate. Image **(D)** shows the RFE-CV, which provided the highest score with the remaining 11 features. Images **(F–H)** show the results of the PCA. Ten recombined dimensions are shown, with the first five explaining 90% of the original data [image **(F)**]. Image g shows the feature distributions of the first five dimensions. We finally selected the features whose contribution was higher than average (the dotted line) on Dimension 1 and Dimension 2 [image **(H)**].

For the T1WI, *t*-tests were first employed to screen out 64 features showing a significant difference between OCH and OSC in the training data. A correlation heat map (Figure 3A) then revealed multicollinearity among the features, and LASSO regression was therefore used to reduce the dimensionality of the features. Figure 3B shows the results of the LASSO regression, and Figure 3C the results of a LASSO-Cross validation (LASSO-CV). Then, 13 features were filtered out and RFE-CV based on an SVM was employed for further feature selection. Figure 3D indicates that the cross validation score reached a peak with 11 features. In view of the limited number of samples, PCA was then applied to these 11 selected features to avoid overfitting due to the large number of features. Figure 3E shows a correlation heat map of the above 11 features and Figures 3F–H the results of the PCA, and eventually, 6 features were selected for building the classification models. Using similar feature reduction methods, 5 features from T2 sequences and 8 features from T1 + T2 sequences were finally selected, and these finally selected features were listed in Table 2.

**Table 2:** The features selected for model construction.

**Sequences**	**Features**
T1	Image_original_Maximum
	**shape_MajorAxisLength**
	**shape_Maximum2DdiameterSlice**
	glcm_JointEnergy
	gldm_DependenceEntropy
	glszm_SmallAreaLowGrayLevelEmphasis
T2	**shape_MajorAxisLength**
	**shape_Maximum2DdiameterSlice**
	glcm_Imc1
	glcm_MCC
	gldm_DependenceEntropy
T1 + T2	**shape_MajorAxisLength**
	**shape_Maximum2DdiameterSlice**
	shape_Maximum2DdiameterColumn
	shape_SurfaceVolumeRatio
	glcm_DifferenceVariance
	glcm_MCC
	glcm_Imc2
	glcm_SumSquares

*Bold font indicates the universal features used for all three sequences. glcm, gray level co-occurrence matrix; gldm, gray level dependence matrix; glszm, gray level size zone matrix; Imc, informational measure of correlation; MCC, maximal correlation coefficient*.

### Model Building and Evaluation

**Figure 4:**
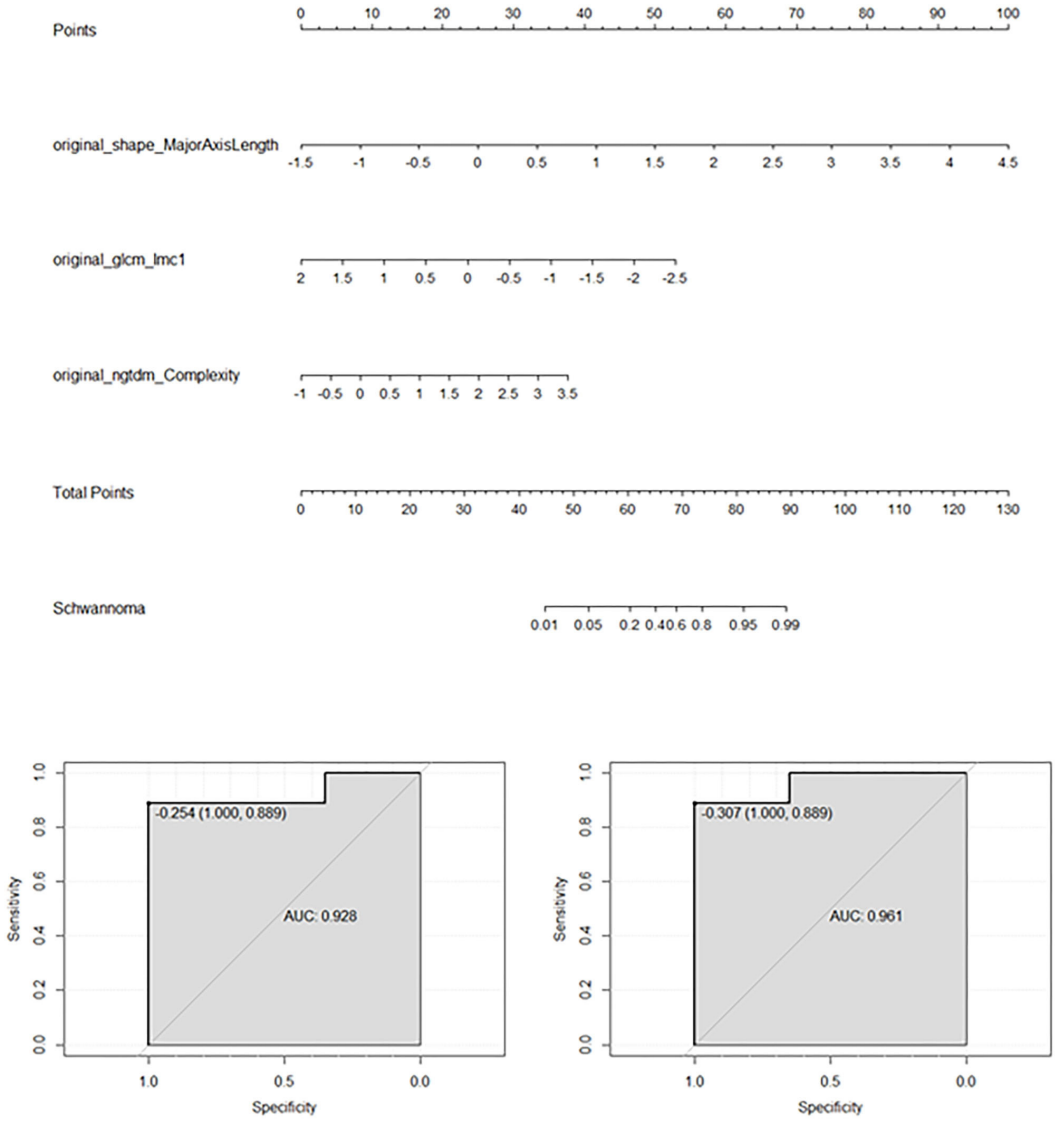
Results of the logistics regression model applied to T2WI. The feature data were standardized with zero-mean normalization. This nomogram is mainly used to predict the odds of OSC rather than OCH according to the three features. The bottom images show the AUCs of the logistic regression model for the training set (left) and validation set (right).

Four models were built using the features described in Table 2. Table 3 shows the accuracy and AUC of each model, and indicates that for all models the T2WI seemed to be superior to the T1WI, both in terms of accuracy and AUC. This table also reveals that the SVM model showed better performance than the other models. Using the T1 + T2 features, the SVM model demonstrated high accuracy of 93% (95% CI: 91–96%) and an AUC of 98% (95% CI: 97–99%). The LR model using T2 features also showed good performance, and a nomogram of this model is exhibited in Figure 4. The LR model had a C-index of 0.93 on the training set and 0.96 on the validation set.

**Table 3:** The ACC and AUC of each model.

**Sequences**	**Models**	**ACC**	**AUC**
T1	LR	77% (67%~83%)	91% (84%~97%)
	SVM	86% (82%~95%)	93% (89%~98%)
	DT	85% (82%~92%)	97% (95%~99%)
	RF	83% (75%~92%)	96% (94%~98%)
T2	**LR**	**93% (85%~100%)**	**91% (89%~92%)**
	**SVM**	**92% (91%~100%)**	**95% (94%~97%)**
	DT	89% (83%~92%)	97% (96%~99%)
	RF	89% (83%~100%)	97% (94%~100%)
T1 + T2	LR	88% (84%~91%)	85% (84%~86%)
	**SVM**	**93% (91%~96%)**	**98% (97%~99%)**
	DT	83% (79%~87%)	96% (95%~97%)
	RF	88% (83%~92%)	97% (95%~98%)

*Models with an ACC over 90% are marked in bold font. ACC, accuracy; AUC, area under the curve; LR, logistic regression; SVM, support vector machine; DT, decision tree; RF, random forest*.

## Discussion

The differentiation of OCH and OSC has long been a clinical problem. In this study, we attempted to solve this problem with radiomics, and the results demonstrated the technique's potential for differentiating between the two disorders.

For orbital tumor patients, T1WI and T2WI are more commonly used than contrast-enhanced MRI, especially for those with renal failure or contrast medium allergy (20). Therefore, our results obtained using only these two routine sequences could be widely used and verified in clinical practice. To explore the contributions of each single sequence and a combined sequence (T1 + T2), we compared radiomics features extracted from each image type. Our results showed that the T2WI provided better accuracy than the T1WI, a conclusion also found in some other studies (16, 21, 22). Hopewell et al. considered that the greater contribution of T2WI may be associated with the heterogeneous angiogenesis of the tumor (23). It is known that ischemia, angioedema, and avascular necrosis are more likely to be observed on T2WI. Furthermore, the much longer echo time on T2WI in comparison with T1WI may also be a factor influencing the results (22). The model using both sequences combined achieved a higher score and AUC than either of the sequences alone, just as Han et al. observed in their studies (16, 24).

OSC may tend to grow rapidly. Of all the selected features, we found that the features of shape_ MajorAxisLength and shape_Maximum2DdiameterSlice were present in all sequence groups. The shape feature MajorAxisLength is defined as the largest axis length of the ROI-enclosing ellipsoid, while the shape feature Maximum2DdiameterSlice is defined as the largest pairwise Euclidean distance between tumor surface mesh vertices in the row-column (generally the axial) plane. As can be seen in Figure 2, a large tumor size may be more indicative of OSC, which may be related to the progressive growth of OSC. Accordingly, a substantial portion of the OCH tumors were found incidentally, whereas almost all the OSC patients were suffering from symptoms of tumor rapid growth. Furthermore, OCH is often found in middle age at 40–50 years, whereas OSC can present at any age (3, 25–27). OSC may manifest heterogeneous on MRI just as a “geographic map.” Except for the above-mentioned two shape features, the other selected features, such as gldm_DependenceEntropy and glcm_MCC, all implied that OSC presented with heterogenous signal intensity. As Tanaka et al. mentioned in their studies (8, 10, 28), OSC is more likely to present heterogeneous than OCH.

The use of radiomics provided a number of advantages compared with previous studies. 1) This was the first attempt to differentiate OCH and OSC with radiomics, and it worked well. 2) Digital features were extracted rather than qualitative assessment through the reader's eye, and detailed data were provided for the analysis rather than a simple statement such as “high signal intensity.” 3) Radiomics can make the reading of images and the analysis automated, thereby reducing human error. 4) Objective and repeatable results are more valuable for the clinician than a subjective judgement. However, there are inevitably some limitations to our study. 1) The limited sample size due to the low incidence of the two disorders may have led to a bias in the results, although we restricted the number of features to avoid overfitting. 2) The data used in this study were from a single center, which may affect the robustness of the model. 3) The absence of an external validation cohort because of the limited samples could also limit the final efficiency. 4) The disproportionate numbers of individuals with OSC and OCH is another limitation, and might have affected the statistics and the results. Therefore, a further multi-center analysis is needed to support our results.

## Data Availability Statement

The raw data supporting the conclusions of this article will be made available by the authors, without undue reservation.

## Ethics Statement

The studies involving human participants were reviewed and approved by Ethics Committee of Shanghai Changzheng Hospital Affiliated with Naval Military Medical University. The patients/participants provided their written informed consent to participate in this study. Written informed consent was obtained from the individual(s) for the publication of any potentially identifiable images or data included in this article.

## Author Contributions

LC: methodology, software, formal analysis, investigation, data curation, writing—original draft, review and editing, and visualization. WY: methodology, writing—review and editing, supervision, and project administration. RW: conceptualization, methodology, validation, investigation, writing—review and editing, supervision, and project administration. JL: data curation and writing—review and editing. HL: resources and data curation. XH: methodology, validation, writing—review and editing. YS: validation, formal analysis, resources, writing—review and editing. All authors contributed to the article and approved the submitted version.

## Funding

This work was supported by National Natural Science Foundation of China (Grant No. 81770959); National Natural Science Foundation of China (Grant No. 81570885).

## Conflict of Interest

The authors declare that the research was conducted in the absence of any commercial or financial relationships that could be construed as a potential conflict of interest.

## Publisher's Note

All claims expressed in this article are solely those of the authors and do not necessarily represent those of their affiliated organizations, or those of the publisher, the editors and the reviewers. Any product that may be evaluated in this article, or claim that may be made by its manufacturer, is not guaranteed or endorsed by the publisher.
